# Supplementary material for: Attractiveness of female sexual signaling predicts differences in female grouping patterns between bonobos and chimpanzees
Source: Commun Biol. 2021 Sep 23;4:1119. doi: 10.1038/s42003-021-02641-w (PMC8460808; doi:10.1038/s42003-021-02641-w)
Supplement: Supplementary file 2 — Supplementary Information [file 42003_2021_2641_MOESM2_ESM.pdf]

Supplementary Information to "Attractiveness of female sexual signaling predicts differences in female grouping patterns between bonobos and chimpanzees"

Supplementary Table 1 shows an overview of the observation time per female in each community. MTF refers to maximally tumescent females.

Female ID	Community	Species	Age (years)	Parity	Focal observation time (h)	Mean number of females in the party	% of observation time with a MTF
F1	Tai_South	Chimpanzee	18-19	Parous	47.5	3.40	31.90
F2	Tai_South	Chimpanzee	13-14	Nulliparous	4	1.00	38.46
F3	Tai_South	Chimpanzee	14-15	Parous	21.5	5.19	40.91
F4	Tai_South	Chimpanzee	46-47	Parous	44.5	2.91	50.00
F5	Tai_South	Chimpanzee	46-47	Parous	37.5	4.98	42.14
F6	Tai_South	Chimpanzee	26-27	Parous	57.5	3.14	51.65
F7	Tai_South	Chimpanzee	17-18	Parous	50	4.63	25.64
F8	Tai_South	Chimpanzee	16-17	Parous	67.5	3.91	36.67
F9	Tai_South	Chimpanzee	17-18	Parous	52	4.60	48.90
F10	Tai_South	Chimpanzee	16-17	Parous	16.5	4.48	18.33
F11	Tai_South	Chimpanzee	51-52	Parous	69	3.07	36.36
F12	Tai_South	Chimpanzee	25-26	Parous	53	3.59	16.67
F13	Tai_South	Chimpanzee	15-16	Parous	54	3.58	29.31
F14	Tai_South	Chimpanzee	26-27	Parous	82	3.09	35.46
F15	Tai_East	Chimpanzee	31-32	Parous	77	3.56	35.83
F16	Tai_East	Chimpanzee	17-18	Parous	72	3.12	23.33
F17	Tai_East	Chimpanzee	26-27	Parous	109.5	3.29	30.23
F18	Tai_East	Chimpanzee	24-25	Parous	84	3.79	80.00
F19	Tai_East	Chimpanzee	36-37	Parous	73.5	2.84	21.56
F20	Tai_East	Chimpanzee	27-28	Parous	58	3.57	29.80
F21	Tai_East	Chimpanzee	16-17	Parous	67.5	3.00	24.94
F22	Tai_East	Chimpanzee	32-33	Parous	33.5	3.42	20.61
F23	Bompusa	Bonobo	21-23	Parous	71.5	3.97	100.00
F24	Bompusa	Bonobo	31-33	Parous	66.5	3.98	18.75
F25	Bompusa	Bonobo	16-18	Parous	56.5	5.47	31.96
F26	Bompusa	Bonobo	31-33	Parous	68.5	5.86	13.61
F27	Bompusa	Bonobo	16-18	Parous	53.5	5.59	3.64
F28	Bompusa	Bonobo	31-33	Parous	61.5	3.96	24.09
F29	Bompusa	Bonobo	31-33	Parous	56	4.18	4.62
F30	Bompusa	Bonobo	13-15	Parous	60.5	4.48	18.77
F31	Bompusa	Bonobo	31-33	Parous	53	5.76	29.63
F32	Bompusa	Bonobo	21-23	Parous	62	2.34	20.20
F33	Bompusa	Bonobo	21-23	Parous	58	4.13	19.28
F34	Bompusa	Bonobo	16-18	Parous	59	2.58	7.44
F35	Bompusa	Bonobo	31-33	Parous	72.5	5.74	0.83

Relationship between time spent feeding and food abundance in bonobos

In order to validate the relationship between time spend feeding and food abundance in bonobos, we used phenological and behavioural data from another bonobo population, Kokolopori, for which detailed information of food availability and feeding behaviour is available for 13 consecutive months[1]. We calculated the monthly proportion of time spend feeding based on group activity scans performed every 10 minutes while following the bonobos (for each scan we scored the activity of the majority of visible individuals; N=12'365). We calculated a *Fruit Abundance Index (FAI)*, which was previously extrapolated for this population (Lucchesi et al., 2020) based on the monthly fruit availability measure of Anderson et al.[2]. We first calculated the monthly FAI (*MFAI*) based on monthly phenological data of bonobo feeding trees and lianas along selected transects, as well as data on tree and liana size and abundance from floristic plots distributed evenly over the home ranges of each community [1]. The calculation is as follows:

$$MFAI = \sum_{i}^{S} P_{im} B_i$$

where P_{im} is the proportion of trees of species i in the phenology trail bearing ripe fruits in month m, B_i is the basal area of species i (i.e., the total cross-sectional area of tree trunks measured at 1.3 m above ground derived from floristic plot data; see above), and S is the total number of species.

We then plotted the MFAI against the proportion of feeding time which revealed a negative correlation with less time spent feeding during times of high food abundance (see Supplementary Figure 1).

Supplementary Figure 1 shows the relationship between time spent feeding by the bonobos and the food abundance index at Kokolopori.

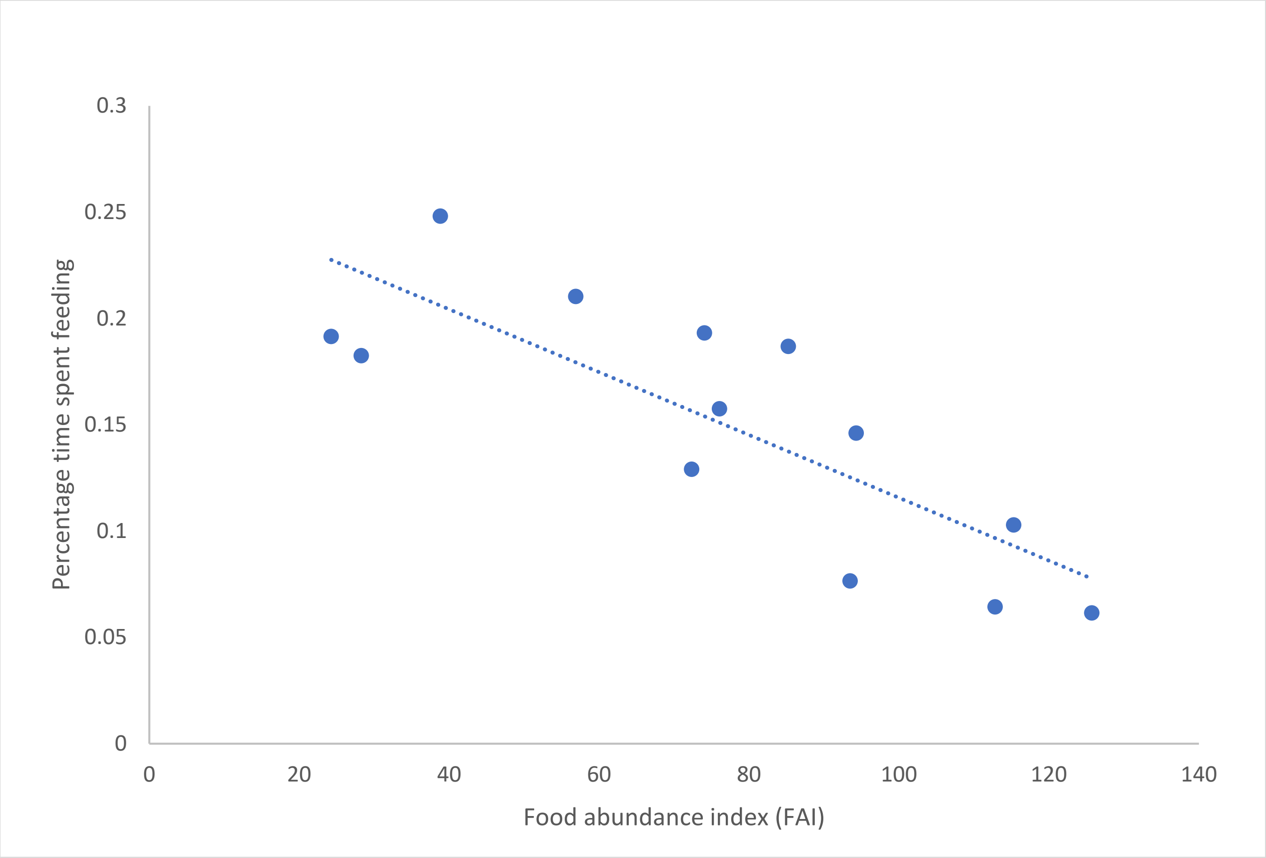

Supplementary Figure 2 shows the average number of party females (a), the average proportion of time spent alone (i.e. with no other adult) (b) and the average proportion of time spent in female only parties (c) for each bonobo (Bompusa) and chimpanzee focal female (Taï South and Taï East). The dots depict the average value for a given female, Triangle symbols indicates the females that had an adult son present in the community. In Taï East, no females had an adult son present in the community.

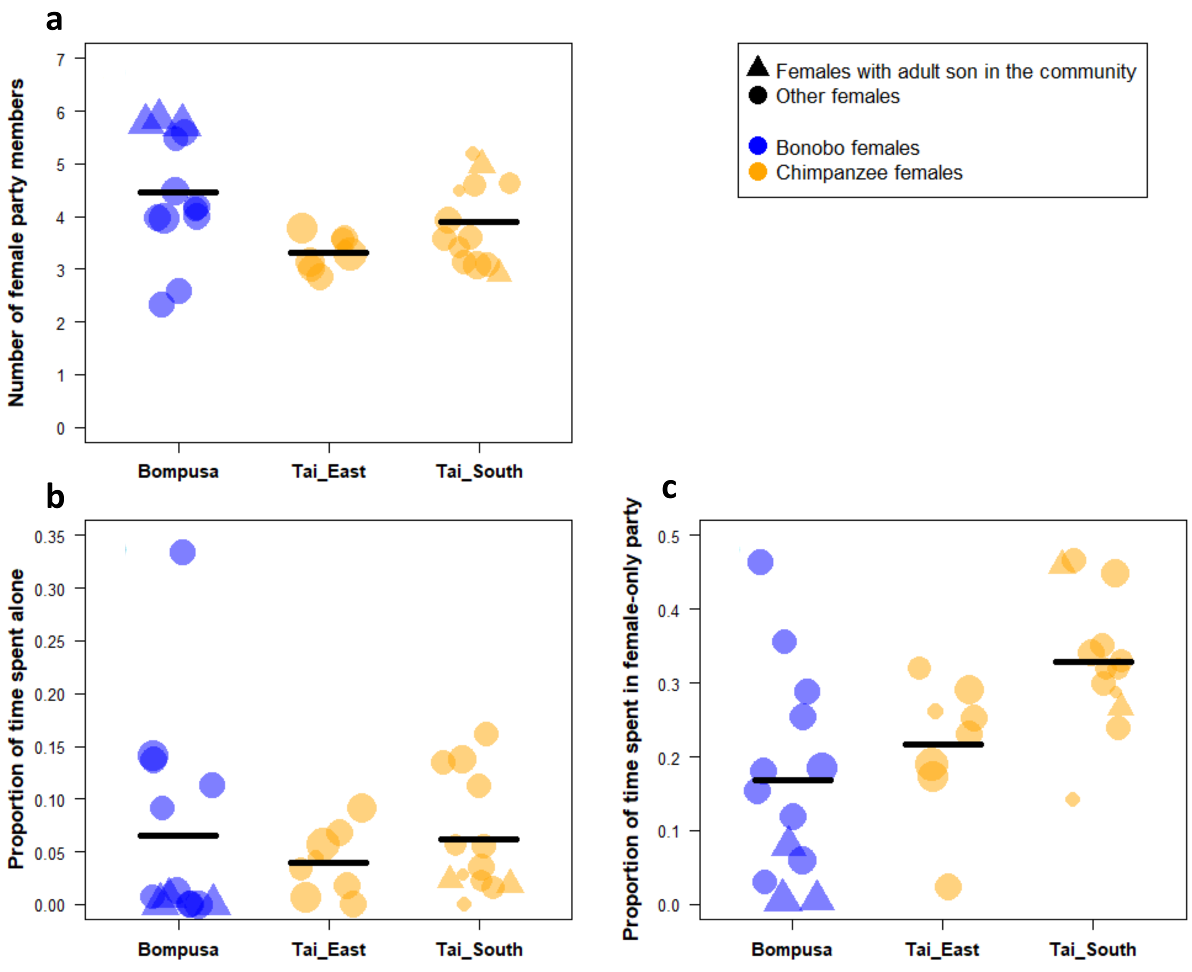

Supplementary Table 2 shows the estimates of a model run on a reduced dataset for the Bompusa bonobo community controlling for food abundance (number of fruiting trees along phenology transects in bonobos and a monthly food abundance index of chimpanzee food items at Taï [3]).

**MODEL**	**Variation in female party sizes in relation to maximally tumescent females (percentage time with maximally tumescent females), using a reduced bonobo dataset and fruit tree data to control for fluctuation in food availability**	
Response	Average number of females in party over the course of a half-day focal follow	
Full-null model	LRT, df=7, $\chi2$= 37.08, P< 0.001	
	Estimate	SE
Intercept	1.04	0.08
Community (Tai East)	0.06	0.10
Community (Tai South)	0.21	0.09
Percentage time with MTF (incl focal)	0.59	0.11
FAI	0.04	0.06
Dominance rank	0.03	0.03
Community (Tai East): Percentage time with MTF (incl focal)	-0.27	0.17
Community (Tai South): Percentage time with MTF (incl focal)	-0.45	0.19
Community (Tai East): FAI	-0.18	0.08
Community (Tai South): FAI	-0.12	0.08
Control	ID/ time of the day	

Supplementary Figure 3 shows the relationship between the monthly proportion of time spent feeding (our behavioural proxy for food abundance) and the number of female party members for female focals. The Bompusa bonobo community is depicted in blue and the two Taï chimpanzee communities are depicted in orange (round dots for Taï_East and diamond dots for Taï_South). Each dot represents a half day focal follow and the size of the dot represents the number of data points (i.e. number of 30min parties) for a given value. The lines indicate the model line controlling for presence of maximally tumescent females, dominance rank, time of the day and multiple sampling of the same individuals in solid blue, solid orange and dashed orange for Bompusa, Taï_East and Taï_South respectively (Model 3b).

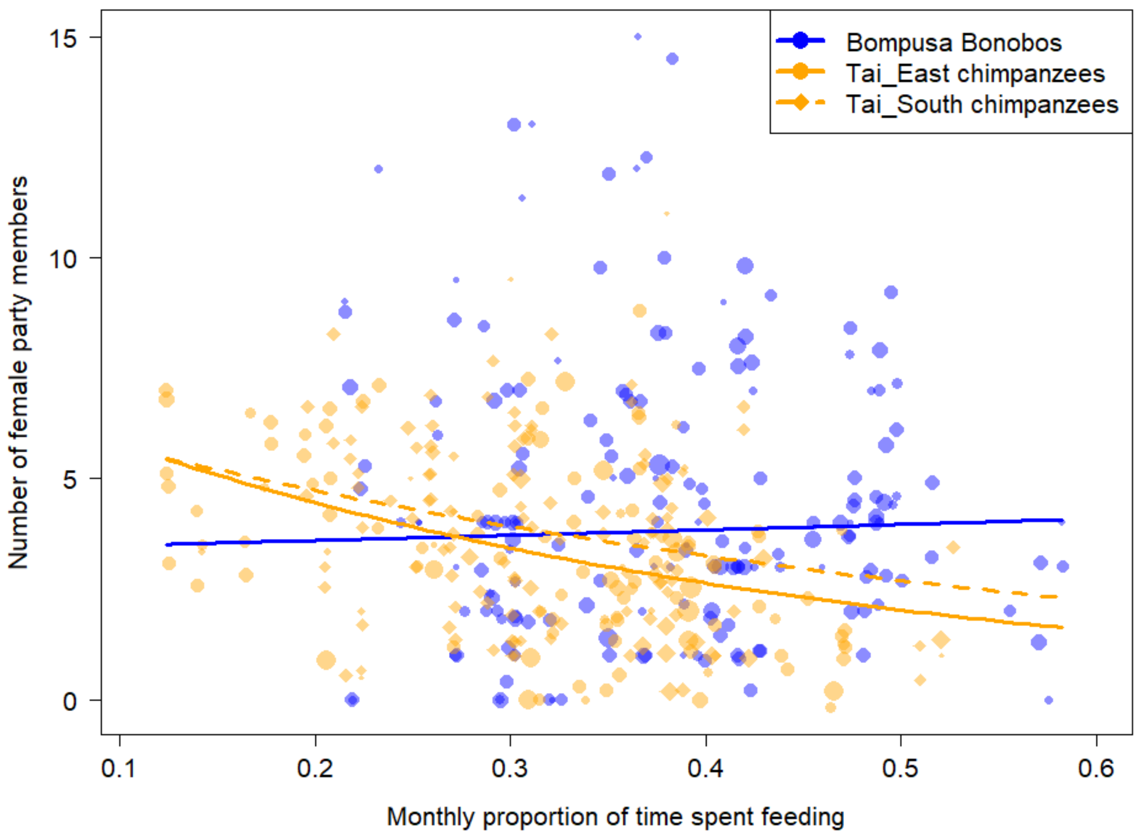

Supplementary Figure 4 shows the average number of party females over the course of our study. The Bompusa bonobo community is depicted in blue and the two Taï chimpanzee communities are depicted in orange and grey. Numbers close to the lines indicate the number of observation hours in a given month.

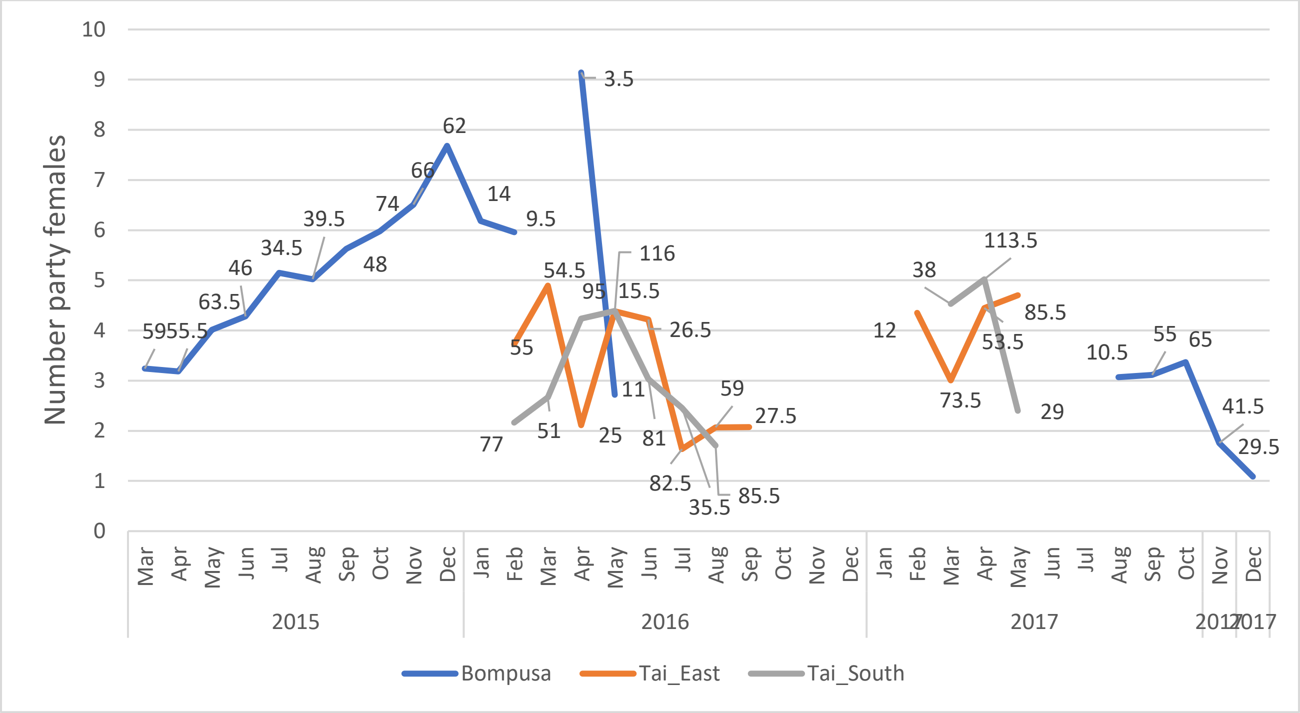

Citations

1 Lucchesi, S., Cheng, L., Janmaat, K., Mundry, R., Pisor, A., & Surbeck, M. (2020). Beyond the group: how food, mates, and group size influence intergroup encounters in wild bonobos. *Behavioral Ecology*, *31*(2), 519–532.

2 Anderson, D. P., Nordheim, E. V., Moermond, T. C., Bi, Z. B. G., & Boesch, C. (2005). Factors influencing tree phenology in Taï National Park, Côte d'Ivoire. *Biotropica*, *37*(4), 631–640.

3 Anderson, D. P., Nordheim, E. V., Boesch, C., & Moermond, T. C. (2002). Factors influencing fission-fusion grouping in chimpanzees in the Taï National Park, Côte d'Ivoire. *Behavioural Diversity in Chimpanzees and Bonobos. Cambridge University Press, Cambridge*, 90–101.
