# Supplementary material for: Attractiveness of female sexual signaling predicts differences in female grouping patterns between bonobos and chimpanzees
Source: Commun Biol. 2021 Sep 23;4:1119. doi: 10.1038/s42003-021-02641-w (PMC8460808; doi:10.1038/s42003-021-02641-w)
Supplement: Supplementary file 3 — Reporting Summary [file 42003_2021_2641_MOESM3_ESM.pdf]

nature research

Corresponding author(s): Martin Surbeck
Cedric Girard-Buttoz

Last updated by author(s): Aug 25, 2021

Reporting Summary

Nature Research wishes to improve the reproducibility of the work that we publish. This form provides structure for consistency and transparency in reporting. For further information on Nature Research policies, see our Editorial Policies and the Editorial Policy Checklist.

Statistics

For all statistical analyses, confirm that the following items are present in the figure legend, table legend, main text, or Methods section.

n/a	Confirmed	
☐	☒	The exact sample size (*n*) for each experimental group/condition, given as a discrete number and unit of measurement
☐	☒	A statement on whether measurements were taken from distinct samples or whether the same sample was measured repeatedly
☐	☒	The statistical test(s) used AND whether they are one- or two-sided<br>*Only common tests should be described solely by name; describe more complex techniques in the Methods section.*
☐	☒	A description of all covariates tested
☐	☒	A description of any assumptions or corrections, such as tests of normality and adjustment for multiple comparisons
☐	☒	A full description of the statistical parameters including central tendency (e.g. means) or other basic estimates (e.g. regression coefficient) AND variation (e.g. standard deviation) or associated estimates of uncertainty (e.g. confidence intervals)
☐	☒	For null hypothesis testing, the test statistic (e.g. *F*, *t*, *r*) with confidence intervals, effect sizes, degrees of freedom and *P* value noted<br>*Give P values as exact values whenever suitable.*
☒	☐	For Bayesian analysis, information on the choice of priors and Markov chain Monte Carlo settings
☒	☐	For hierarchical and complex designs, identification of the appropriate level for tests and full reporting of outcomes
☐	☒	Estimates of effect sizes (e.g. Cohen's *d*, Pearson's *r*), indicating how they were calculated

Our web collection on statistics for biologists contains articles on many of the points above.

Software and code

Policy information about availability of computer code

Data collection	Cybertracker
Data analysis	R 3.6.1 (R Core Team 2019)

For manuscripts utilizing custom algorithms or software that are central to the research but not yet described in published literature, software must be made available to editors and reviewers. We strongly encourage code deposition in a community repository (e.g. GitHub). See the Nature Research guidelines for submitting code & software for further information.

Data

Policy information about availability of data

All manuscripts must include a data availability statement. This statement should provide the following information, where applicable:

- Accession codes, unique identifiers, or web links for publicly available datasets
- A list of figures that have associated raw data
- A description of any restrictions on data availability

The datasets and the codes for the analysis in the current study are available from the corresponding author on reasonable request

Field-specific reporting

Please select the one below that is the best fit for your research. If you are not sure, read the appropriate sections before making your selection.

☐ Life sciences ☒ Behavioural & social sciences ☐ Ecological, evolutionary & environmental sciences

For a reference copy of the document with all sections, see nature.com/documents/nr-reporting-summary-flat.pdf

Behavioural & social sciences study design

All studies must disclose on these points even when the disclosure is negative.

Study description	This is a observational study relying on quantitative data collected in the field.
Research sample	We included all adult parous females at the start of our study period as our focal individuals (estimated older than13 years). The East community of Taï chimpanzee consisted of 14 adult individuals (5 males, 9 females). One of the adult females could not be followed individually due to her shyness towards observers and was not included in the party composition. None of the females in this community had an adult son present. The South community of Taï chimpanzee consisted of 18-20 adult individuals (5-6 males and 13-14 females). One female and a male disappeared during the course of the study. Two females in this community had an adult son present. Finally, the Bompusa community consisted of 20-21 adult individuals (7 males and 14-15 females, one of which immigrated into the study community after the start of the study period and was not followed as a focal individual but included in the party composition. In addition, one extra female was a temporary visitor and not included in the analysis (see SI Table 1 for details on the age and observation hours of each focal female). Three females in this community had an adult son present.
Sampling strategy	NA
Data collection	We relied on female focal individual follows and all data collector used the same interface of the software Cybertracker on an android smartphone
Timing	We conducted the study at the field sites of LuiKotale, DRC (Fruth & Hohmann 2018) and Taï, Côte d'Ivoire (Wittig 2018), between February 2015 and December 2017 (for exact time span of data collection periods at each site see Methods section of the manuscript)
Data exclusions	NA
Non-participation	NA
Randomization	NA

Reporting for specific materials, systems and methods

We require information from authors about some types of materials, experimental systems and methods used in many studies. Here, indicate whether each material, system or method listed is relevant to your study. If you are not sure if a list item applies to your research, read the appropriate section before selecting a response.

Materials & experimental systems

n/a	Involved in the study	
☒	☐	Antibodies
☒	☐	Eukaryotic cell lines
☒	☐	Palaeontology and archaeology
☐	☒	Animals and other organisms
☒	☐	Human research participants
☒	☐	Clinical data
☒	☐	Dual use research of concern

Methods

n/a	Involved in the study	
☒	☐	ChIP-seq
☒	☐	Flow cytometry
☒	☐	MRI-based neuroimaging

Animals and other organisms

Policy information about studies involving animals; ARRIVE guidelines recommended for reporting animal research

Laboratory animals	NA
Wild animals	The study included only non-invasive observation of wild living animals.
Field-collected samples	NA

Ethics oversight	Ethical guidelines adhere to those defined by the former department of Primatology at the Max Planck Institute for Evolutionary Anthropology: https://www.eva.mpg.de/primat/ethical-guidelines/?Fsize=0%252527&cHash=d70e8adae0f98580648bf02d09db18d0

Note that full information on the approval of the study protocol must also be provided in the manuscript.
